# Preventing regrettable substitutions: a proposal for a tiered testing approach for BPA alternatives under the PARC initiative

**DOI:** 10.3389/ftox.2026.1815869

**Published:** 2026-07-09

**Authors:** Kiara Aiello-Holden, Kristina Bartmann, Franca Maria Buratti, Nicolas J. Cabaton, Lucia Coppola, Emanuela Corsini, Antonio De la Vieja, Hubert Dirven, Maria Dusinska, Naouale El Yamani, Nina Franko, Johanna M. Gostner, Nathalie Grova, Louise Gütter, Patricia Iglesias-Hernández, Martina Iulini, Anja Kodila, Yvonne Kohl, Gabriele Lori, Henriqueta Louro, Francesca Marcon, Sakina Mhaouty-Kodja, Archibold Mposhi, Lydie Naulé, Simonetta Palleschi, Barbara Rossi, Elise Rundén-Pran, Francesca Salani, Nicoletta Santori, Maria João Silva, Marija Sollner Dolenc, Emanuela Testai, Mónica Torres-Ruiz, Jonathan Turner, Catherine Viguié, Daniel Zalko, Sabrina Tait

**Affiliations:** 1 German Federal Institute for Risk Assessment (BfR), Berlin, Germany; 2 IUF - Leibniz Research Institute for Environmental Medicine, Düsseldorf, Germany; 3 Biomarkers and Models Unit, Department Environment and Health, Istituto Superiore di Sanità, Mechanisms, Rome, Italy; 4 Toxalim: Research Center in Food Toxicology, INRAE, Université de Toulouse, Toulouse, France; 5 Center for Gender‐Specific Medicine, Istituto Superiore di Sanità, Rome, Italy; 6 Department of Pharmacological and Biomolecular Sciences ‘Rodolfo Paoletti’, Université degli Studi di Milano, Milan, Italy; 7 Endocrine Tumor Unit from Chronic Disease Program (UFIEC), Instituto de Salud Carlos III (ISCIII), Madrid, Spain; 8 Division of Climate and the Environment, Department of Chemical Toxicology, Norwegian Institute of Public Health, Oslo, Norway; 9 Health Effects Laboratory, Department for Environmental Chemistry, NILU-Norwegian Institute for Air Research, Kjeller, Norway; 10 Faculty of Pharmacy, University of Ljubljana, Ljubljana, Slovenia; 11 Institute for Medical Biochemistry and Core Facility Metabolomics II, Medical University of Innsbruck, Innsbruck, Austria; 12 Immune Endocrine Epigenetics Research Group, Department of Infection and Immunity, Luxembourg Institute of Health, Esch-SurAlzette, Luxembourg; 13 Fraunhofer Institute for Biomedical Engineering IBMT, Sulzbach, Germany; 14 Department of Human Genetics, National Institute of Health Dr. Ricardo Jorge, Lisbon, Portugal; 15 Comprehensive Health Research Centre (CHRC), NOVA Medical School, Universidade NOVA de Lisboa, Lisbon, Portugal; 16 Development, Adaptation and Aging, Sorbonne Université, CNRS, INSERM, Paris, France

**Keywords:** bisphenol a alternatives, carcinogenicity, developmental neurotoxicity (DNT), endocrine disruption (ED), human hazard assessment, immunotoxicity, metabolism, new approach methodologies (NAMs)

## Abstract

Bisphenol A (BPA) alternatives are increasingly used in the manufacture of industrial and consumer products, following regulatory restrictions on BPA. However, insufficient safety data on these substitutes raise concern as regards potential regrettable substitutions. Under the EU Partnership for the Assessment of Risks from Chemicals (PARC), Work Package 5 (WP5) addresses this challenge by applying a human-relevant tiered hazard assessment strategy grounded on OECD test guidelines as first tier and expanding the battery to include NAMs (New Approach Methodologies). Eight BPA alternatives were prioritized for studies addressing key toxicological endpoints, namely, endocrine disruption (ED), developmental neurotoxicity (DNT), immunotoxicity, genotoxicity and carcinogenicity and metabolic fate examination (detoxification vs. potential bioactivation), to enable early identification of biological activity and support cross-endpoint prioritization. This manuscript describes the structure and implementation of the testing framework. This integrated testing strategy proposes a structured approach to identify substances of potential concern, guide targeted higher-tier studies, and support regulatory prioritization. PARC WP5 framework is testing whether coordinated NAM-based methods may contribute to next-generation risk assessment and help prevent regrettable substitutions among BPA alternatives for rapid regulatory adoption. Detailed experimental results will be reported separately upon completion of the project.

## 1 Introduction

The widespread use and subsequent regulatory restriction of Bisphenol A (BPA) have accelerated the development and use of alternative substances. Although some alternatives, such as Bisphenol S (BPS), have been thoroughly examined, toxicological data is still insufficient for many other BPA alternatives. This situation raises the risk of *regrettable substitutions*, where one hazardous substance is replaced with another of similar or greater concern.

In the framework of the EU Partnership for the Assessment of Risks from Chemicals (PARC), a Project within Work Package 5 (WP5) aims to fill critical data gaps for eight BPA alternatives: BPZ, BPE, BPS-MAE, Pergafast 201, BPP, BPAP, TCBPA, TBBPA. The compounds were prioritized following discussion with experts from involved regulatory agencies as the European Food Safety Authority (EFSA) and the European Chemical Agency (ECHA) (Aiello Holden et al., 2023; Mhaouty-Kodja et al., 2024). This project focuses on generating human health-relevant data across five toxicological endpoints, based on BPA-induced health effects and their regulatory relevance: endocrine disruption (ED), developmental neurotoxicity (DNT), immunotoxicity, genotoxicity and carcinogenicity, and metabolic fate examination (detoxification vs. potential bioactivation).

Beyond generating endpoint-specific hazard data, the PARC WP5 initiative aims to operationalize and integrate NAM-based testing architecture that supports regulatory prioritization of structurally related chemical alternatives (Figure 2). By combining harmonized experimental design, centralized substance procurement, and cross-endpoint interpretation strategies, the project will provide a proof-of-concept for how large-scale European collaborations can implement next-generation risk assessment (NGRA) principles in practice (Herzler et al., 2024; 2025; Marx-Stoelting et al., 2023).

While experimental work is ongoing across endpoints, this manuscript focuses on the design, coordination, and implementation of the tiered NAM-based testing framework. Detailed experimental results will be presented in subsequent publications, expected during the first quarter of 2027.

## 2 Project strategy and tiered assessment framework

**FIGURE 1:**
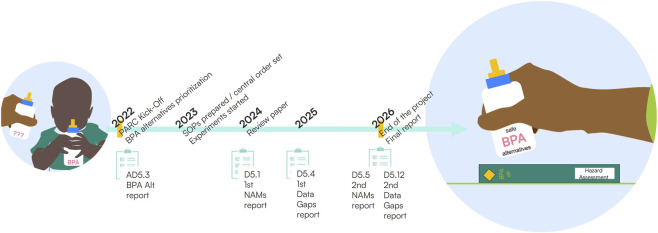
Timeline of the PARC WP5 project for the hazard assessment of BPA alternatives. Project started May 2022 and will finalize October 2026. Results per endpoint will be reported in the PARC Deliverable Data Gaps report (Submitted to HaDEA, EU Commission for revision the last quarter of 2026, and publicly available 2027). All deliverables can be found in the PARC website: https://www.eu-parc.eu/deliverables. Integrated analysis of the generated datasets and publication of detailed experimental results are expected during the first quarter of 2027.

A carefully planned and agreed strategy is followed to ensure that integration of data from the several toxicological endpoints will be possible and useful for scientific and regulatory purposes. The strategic planning is shown in Figure 1 and included the following critical points:

- Tested Compounds:

Eight prioritized BPA alternatives and BPA as reference compound (Table 1).

- Selection Process:

**TABLE 1:** Prioritized BPA Alternatives for human hazard assessment.

Substance	Abbreviation	CAS no.	Molecular weight (g/mol)	Structure	Consumer and industrial applications
Bisphenol A	BPA	80–05–7	228,29	C_15_H_16_O_2_	Broad use, including epoxy resins and polycarbonate plastics (industrial and consumer goods: e.g., lacquers, plastics), thermal printing papers
Bisphenol Z	BPZ	843–55–0	268,35	C_18_H_20_O_2_	Industrial intermediate and monomer used in resins, coatings, and specialty polymer applications
Bisphenol E	BPE	2081–08–5	214,26	C_14_H_14_O_2_	Coatings, adhesives, and plastic additives
Bisphenol S-MAE	BPS-MAE	97,042–18–7	290,34	C_19_H_16_O_4_S	Polymers, coatings, sealants, thermal printing papers
Pergafast 201	PF201	232,938–43–1	460,52	C_21_H_20_N_2_O_6_S_2_	Used specifically in thermal printing papers
Bisphenol P	BPP	2167–51–3	346,46	C_24_H_26_O_2_	Resins, adhesives, and plasticizers, potential uses in electronics and composites
Bisphenol AP	BPAP	1571–75–1	290,36	C_20_H_18_O_2_	Thermal papers, plastics, and some food contact materials
Tetrachloro bisphenol A	TCBPA	79–95–8	366,07	C_15_H_12_Cl_4_O_2_	Flame retardant in plastics and epoxy resins
Tetrabromo bisphenol A	TBBPA	79–94–7	543,88	C_15_H_12_Br_4_O_2_	Flame retardant in electronics, particularly in printed circuit boards (flame proof epoxy resins)

Substance prioritization was co-designed with input from experts across endpoints and representatives from regulatory agencies (e.g., EFSA, ECHA) by the 17th June 2022. All details can be found in this Additional Deliverable 5.1 (Aiello Holden et al., 2023).

- Centralized Procurement:

All compounds were centrally ordered and distributed to ensure batch consistency and facilitate inter-laboratory comparability.

### Tiered approach

**FIGURE 2:**
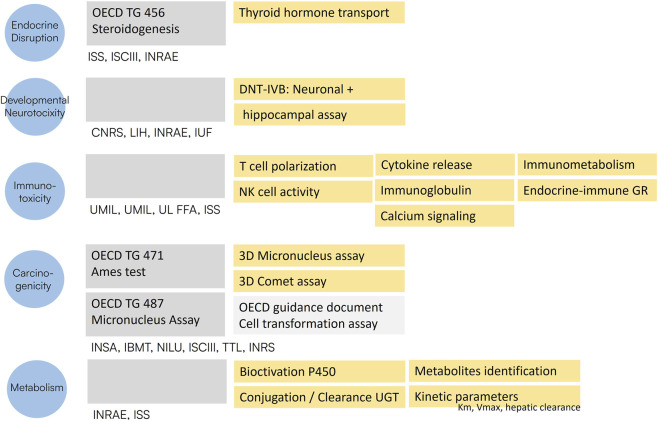
NAM-based strategy to address data gaps for BPA alternatives. Integrated NAM-based testing framework applied to BPA alternatives across endocrine, neurodevelopmental, immune, carcinogenic, and metabolic endpoints. Grey boxes indicate OECD Test Guideline, whereas yellow boxes represent complementary mechanistic NAM approaches supporting cross-endpoint prioritization.

A harmonized and stepwise testing strategy is used, starting with OECD TG-aligned *in vitro assays* and advancing toward more complex studies *in vitro/in silico* assays per endpoints as detailed in the following sections (Figure 2).

### 2.1 Endocrine disruption

The endocrine activity of BPA and the eight prioritized BPA alternatives has been recently reviewed by WP5 partners (Mhaouty-Kodja et al., 2024), highlighting the knowledge gaps about these substitutes. Endocrine disruption represents a critical endpoint in the evaluation of BPA alternatives, as regulatory restrictions on BPA originated from its estrogenic properties. In addition, BPA has been shown to interfere with other endocrine pathways, including thyroid hormone regulation (Mhaouty-Kodja et al., 2024). Therefore, the present project within PARC WP5 investigates endocrine-related effects of these compounds using a tiered battery of OECD guideline–compliant and mechanistically informative *in vitro* assays targeting both steroidogenesis and thyroid hormone–related pathways.

#### 2.1.1 Steroidogenesis (OECD TG 456)

The OECD TG 456 is used to assess alteration of steroidogenesis induced by the eight prioritized BPA alternatives, using BPA as a reference. The assay is based on the use of the human adrenocortical carcinoma cell line H295R, which expresses all the enzymes of this pathway (OECD, 2025). Cytotoxicity testing is first performed to identify concentrations decreasing cell vitality above a 20% threshold value. Then, according to the TG, testosterone and 17β-oestradiol levels are measured after chemical exposure at concentrations below the 20% cytotoxicity threshold. Progesterone, aldosterone and cortisol levels are also measured.

#### 2.1.2 Thyroid hormone transport and iodide uptake

Because thyroid-related disruption is not fully covered by currently adopted OECD Test Guidelines, the PARC WP5 endocrine-disruption battery also includes mechanistically informative NAMs addressing early key events of thyroid hormone homeostasis. Specifically, the project evaluates interference with triiodothyronine (T3) transport through monocarboxylate transporter 8 (MCT8) and iodide uptake through the sodium/iodide symporter (NIS), two essential processes for intracellular thyroid hormone availability and thyroid hormone biosynthesis. Although these assays are not yet adopted OECD Test Guidelines, both the MCT8-and NIS-based Sandell–Kolthoff methods are included in the EURL ECVAM/TSAR thyroid method portfolio as methods selected for thyroid validation activities, and they are aligned with the OECD/EU-NETVAL efforts to evaluate *in vitro* methods for thyroid disruption (European Commission Joint Research Centre, 2019; OECD, 2024). Their inclusion therefore provides mechanistic information on thyroid-related modes of action not captured by guideline-based first-tier assays.

Functional transport assays are performed in MDCK cells stably overexpressing human MCT8 or human NIS, using the corresponding parental cell lines to correct for basal uptake. BPA and the prioritized BPA alternatives are tested across concentration-response ranges, together with reference inhibitors as positive controls. Transport activity is analysed using complementary descriptors, including IC_50_, LOEC and BMD, allowing both classical inhibitory potency and early biologically relevant deviations from control activity to be assessed.

To complement these transporter-based assays, thyroid-cell-specific responses are investigated in the TSH-dependent rat thyroid follicular cell line PCCl3. Cellular proliferation and viability are assessed to distinguish thyroid-related molecular effects from non-specific cytotoxicity. In selected representative compounds, including BPA and several BPA alternatives, acute and prolonged exposures are further used to analyse genes involved in iodide uptake, thyroid differentiation and function, thyroid hormone synthesis/metabolism, and oxidative-stress responses. In addition, LC-MS/MS measurements are used to verify actual exposure concentrations in culture medium and to estimate the apparent cell-associated fraction of selected bisphenols.

Overall, this thyroid module is designed to capture potential differences in biological activity among BPA alternatives, supporting their prioritization for further evaluation. Several compounds interfere with MCT8-mediated T3 transport and/or NIS-mediated iodide uptake, with different relative potencies depending on the transporter. Selected alternatives also induce compound-specific transcriptional signatures in thyroid cells, including changes in genes related to iodide transport, thyroid hormone biosynthesis/metabolism and oxidative-stress adaptation, generally without marked loss of viability under the mechanistic follow-up conditions. Together, these data support the value of combining transporter-based thyroid NAMs with thyroid-cell molecular endpoints to prioritize BPA alternatives for higher-tier testing and avoid assuming safety based solely on structural substitution.

### 2.2 Developmental neurotoxicity

DNT is a critical endpoint for BPA alternatives due to evidence linking BPA to neurodevelopmental effects including on learning and memory processes (European Commission, 2016; European Chemicals Agency, 2017; Mhaouty-Kodja et al., 2018). As an alternative approach to *in vivo* DNT studies and in compliance with the 3R rules of animal experimentation, WP5 is applying a tiered strategy to detect early neurodevelopmental disturbances *in vitro*, in line with the ongoing efforts to develop an *in vitro* test battery by OECD and EFSA (OECD, 2023a).

Standard assays from the DNT *in vitro* testing battery (IVB) (OECD, 2023b) is applied using human neural progenitor cells (hNPCs) to assess proliferation (3-day exposure), neuronal and oligodendrocyte differentiation, neurite outgrowth (5-day exposure), and synaptogenesis (14-day exposure) to BPA and the seven prioritized alternatives (Table 1). Preliminary screening included viability assessment to define concentration ranges for subsequent mechanistic studies.

The DNT-IVB lacks endpoints for hippocampal differentiation and related cellular processes. Therefore, to complement the core *in vitro* test battery and enhance human relevance, a novel assay addressing hippocampal neuron differentiation and maturation, is being developed and qualified. This extension aims to capture region-specific effects related to processes critical for learning and memory not addressed by existing efforts.

The analyses will involve the exposure of hippocampal neurons derived from human induced pluripotent cells to BPA alternatives for 14 days. Cellular viability will be analyzed to determine the non-cytotoxic doses to use in the following experiments addressing neuronal maturation and neuronal network formation, as well as gene expression of synaptic plasticity markers and key hormonal pathways including sex steroid and thyroid hormone receptors. Finally, given that BPA was shown to modify expression of target genes through modifications in DNA methylation (Kundakovic et al., 2013), analyses of global DNA methylation will be conducted.

The hippocampal differentiation assay is currently at an early development stage based on established human iPSC-derived cell systems; therefore, formal readiness scoring is still premature. In parallel, the more mature DNT-IVB assay are currently undergoing inter-laboratory transfer activities across partners in Europe and the USA, supported by EFSA-related initiatives.

### 2.3 Immunotoxicity

The immune system is a sensitive target for chemical exposure, and immunotoxicity assessment is gaining importance in the regulatory evaluation of endocrine-active and environmentally persistent substances (Kaplan et al., 2019). BPA and its analogues have the potential to disrupt or modulate immune function, potentially increasing susceptibility to infections and altering inflammatory responses as recently reviewed (Mhaouty-Kodja et al., 2024). More importantly, the immune system was identified by EFSA as the most sensitive to BPA exposure (Lambré et al., 2023). The critical endpoint leading to this revision was the increase in T helper (Th) 17 cells and IL-17, which are linked to inflammation in autoimmune disease, increased risk of airway inflammation, allergic diseases, and asthma (Lambré et al., 2023). This makes central the evaluation of the effects of BPA analogues on the immune system. Within WP5, immunotoxic potential of BPA alternatives is explored through *in silico*, and *in vitro* assays. For the *in vitro* tests, preference is given to the use of primary peripheral blood mononuclear cells obtained from healthy donors and human-derived cell lines (e.g., THP-1, Jurkat T cells, lymphoblastoid cell lines) (Johnson et al., 2025). After determination of (non)toxic concentrations, the following activities are undertaken:

#### 2.3.1 Functional assessment of immune responses

- T cells polarization and NK cell activity: Effects on T-cell differentiation (Th1, Th2, Th17, Treg) and NK-cell lytic activity are evaluated.
- Cytokine release and immunoglobulin production: The ability of BPA analogues to influence cytokine (IL-1β, IL-2, IL-4, IL-6, IL-10, IL-17A, IFN-γ, TNF-α) and immunoglobulin (IgG, IgM) production is evaluated at concentrations that are not cytotoxic.

#### 2.3.2 Metabolic aspects of immunomodulation

The cellular metabolic activity in PBMCs and monocyte-derived cell lines exposed to BPA analogues is assessed using resazurin. This reports cellular reductive capacity and serves as an indirect marker of viability. In addition, effects of BPA and substitutes on inflammation-driven tryptophan (Trp) catabolism are evaluated by measuring kynurenine (Kyn) formation. Kyn/Trp serves as a surrogate marker of indoleamine 2,3-dioxygenase-1 (IDO-1) activity (Gostner et al., 2015).

#### 2.3.3 Endocrine–immune crosstalk

To provide mechanistic insight into observed immunomodulatory effects and link them to endocrine disrupting effects, an *in silico* screening of selected compounds binding affinity to nuclear receptors is performed. Specifically, by using the freely available platform Endocrine Disruptome, the binding affinity of BPA substitutes to androgen receptor (AR), estrogen receptors α (ERα) and β (ERβ), glucocorticoid receptor (GR), liver X receptors α (LXRα) and β (LXRβ), mineralocorticoid receptor (MR), peroxisome proliferator-activated receptors α (PPARα), β (PPARβ) and γ (PPARγ), progesterone receptor (PR), retinoid X receptor α (RXRα), thyroid receptors α (TRα) and β (TRβ) is predicted. The most potent compounds will be also subjected to *in vitro* immunomodulatory assays.

- Glucocorticoid Receptor (GR) Interactions: BPA analogues' interactions with the glucocorticoid receptor (GR) are studied by measurement of affinity to GR (binding studies) and *in vitro* transactivation assays (using luciferase reporter assays).
- Gene Expression Modulation: The impact of GR activation or suppression on downstream genes is confirmed using qPCR assays in immune cell lines.

#### 2.3.4 Mechanistic and pathway analysis

- Calcium signalling: BPA analogue effects on resting calcium levels and TCR-induced calcium responses are assessed in Jurkat T cells. Changes in calcium signaling, a key event in T cell activation, are analyzed to identify potential molecular initiating events linked to immunotoxicity.

The combined *in silico* and *in vitro* approaches will provide a robust framework for identifying the immunotoxic potential of BPA analogues. The use of human-relevant models and assays targeting key immune functions, such as cytokine regulation, T cell polarization, and antigen-presenting cell activity, enables the detection of subtle immune-modulating effects that may not emerge in standard toxicity testing. The integration of mechanistic endpoints, including alterations in calcium signalling, glucocorticoid receptor interactions, and tryptophan metabolism, further enhances the capacity to link molecular events to adverse immune outcomes. Overall, this integrated approach highlights the potential of combining functional and mechanistic endpoints to assess immunotoxicity and support AOP development.

### 2.4 Genotoxicity and carcinogenicity

Genotoxicity and carcinogenicity testing play a central role in the hazard identification and risk assessment of chemicals. Under the EU chemicals regulatory frameworks, genotoxicity assessment generally requires a battery of tests covering key endpoints for carcinogenesis, including gene mutation, numerical and structural chromosomal aberrations (EFSA Scientific Committee, 2011). Although carcinogenicity assessment generally relies on long-term rodent bioassays, several limitations and ethical concerns have been raised, leading to the development of New Approach Methodologies (Bossa et al., 2026), including those based on oncotransformation of normal cells.

BPA has been linked to carcinogenic effects in both estrogen-sensitive and -insensitive tissues, e.g., mammary gland and liver, respectively. It is unlikely that BPA acts as a direct genotoxicant (Lambré et al., 2023), but several evidences have shown that it may induce secondary genotoxic effects, mediated by reactive oxygen species induction and non-genotoxic effects, particularly, through epigenetic modification that dysregulate the expression of critical cancer-related genes (Mhaouty-Kodja et al., 2024; Cimmino et al., 2020). Emerging evidence suggests that certain BPA alternatives also display characteristics of a carcinogen, including the ability to induce genotoxicity, epigenetic dysregulation, and increased cell proliferation (Mhaouty-Kodja et al., 2024), underscoring the need for further studies to assess their potential cancer risk.

In this WP5 initiative, a tiered strategy is applied in order to build a weight-of-evidence evaluation of the selected BPA alternatives’ genotoxic and non-genotoxic mode of action, aligned with OECD guidance to detect early indicators of carcinogenic potential.

In a first tier, the primary battery of tests includes the *in vitro* bacterial reverse mutation test (Ames test) following the OECD Test Guideline 471 (TG471; OECD, 2020), the *in vitro* micronucleus (MN) test in human lymphocytes (TG487; OECD, 2023a), and the cell transformation assay (CTA) with Bhas-42 cells (GD231; OECD, 2016) (Figure 2). The concentration-range of each test substance is defined based on its preliminary cytotoxicity or insolubility, according to the respective TG/GD. The cytotoxicity will be assessed either using colorimetric assays (e.g., MTT assay) or using the cytokinesis-block proliferation index (CBPI) determined as part of the MN assay (OECD, 2023b).

The results are intended to provide information on whether each tested BPA alternative is able to produce gene mutations, structural and numerical chromosome aberrations or to induce morphological transformation of mammalian cells, which are hallmarks of a carcinogenic potential (Smith et al., 2016). The addition of the CTA to the genotoxicity tests battery allows the detection of changes associated with tumor initiation and progression in an *in vitro* system, which is useful when *in vivo* carcinogenicity data are not yet available and, together with the results from the genotoxicity tests, are expected to reduce animal experimentation. The results of the CTA may also aid on the interpretation of potentially conflicting genotoxicity results.

In a second tier, extended mammalian cell genotoxicity assays are conducted using the human hepatocellular carcinoma cell line HepG2 in bi-dimensional (2D) and tri-dimensional cultures (3D, spheroids). The results of the comet and the MN assays in 2D cultures are expected to give supporting evidence as to the genotoxic potential of the selected BPA alternatives. Furthermore, the use of spheroids increases human relevance beyond standard OECD TGs to characterize the potential effects of the chemicals on DNA and chromosome damage in a partial metabolism competent cell line. A second aim is to comparatively analyse the results of genotoxicity testing in 2D and 3D cultures, the latter being a NAM that improves tissue relevance. To support assay transferability and future regulatory uptake, the 3D spheroids assay was assessed using the ReadEDTest framework (Crouzet et al., 2023), achieving a high readiness score (33.5/38), indicating strong laboratory transferability and suitability for future formal validation activities.

The WP5 Project prioritizes the above tiered set to maximize human cellular relevance and ensure cross-endpoint integration (with metabolism and DNT). The integration of the three *in vitro* assays in tier one contributes to close data gaps and support regulators to reach a conclusion on whether each tested BPA alternative may pose a carcinogenic risk. Based on the results of the proposed tests’ combination and the consistency across assays it can also be decided if further *in vitro* testing is needed (e.g., a gene mutation assay in mammalian cells) prior to *in vivo* testing. This strategy aligns with ECHA/EFSA guidance, which encourages the integration of *in vitro* assays to refine or eventually replace *in vivo* genotoxicity studies. With tier two assays, by integrating genotoxicity analyses in a 3D model, more robust insights into the genotoxic and carcinogenic potential of the test substances and associated mechanisms are expected, filling critical data gaps and supporting prioritization of BPA alternatives for higher-tier TG + studies and reducing reliance on animal testing.

### 2.5 Metabolic fate examination

Understanding the metabolic fate and potential bioactivation/detoxification routes of BPA alternatives is key for interpreting related toxicological data, and pave the road for extrapolating *in vitro* data to *in vivo* scenarios. This action focuses on examining whether biological activity/toxicity arises from the parent compound or from the formation of one or several metabolite(s). The metabolic fate of many BPA alternatives remains largely unexplored, notably in human *in vitro* models, as previously reviewed (Mhaouty-Kodja et al., 2024). Studies span from physicochemical characterization (solubility, bioavailability) to the assessment of Phase I and Phase II enzymatic activities involved in BPA alternatives biotransformation.

#### 2.5.1 Substance stability and solubility harmonization

A harmonized SOP was developed and distributed across laboratories to standardize stock preparation, solubility testing, and dilution procedures, so to ensure comparable exposure conditions across assays, forming the basis for assessing substances stability and bioavailability in cell culture media. In addition to reference (unlabelled) standards, radiolabelled molecules are purchased or synthesized for most studied alternatives, to back-up solubility and stability studies, and allow further exploration of their biotic (and abiotic) transformation.

#### 2.5.2 Phase I metabolism and bioactivation

Human hepatic models (HepaRG and HepG2 cells), human sub-cellular fractions (liver, kidney and intestine; microsomal fractions) and other tools (CYP specific supersomes) are used to characterize the fate of the test substances, including via oxidative metabolic routes mediated by cytochrome P450 (CYP) enzymes.

#### 2.5.3 Phase II conjugation and clearance

Glucuronidation and sulfation pathways, which are pivotal in the detoxification metabolism of bisphenols are also investigated using human sub-cellular fractions (liver, kidney and intestine; microsomal and/or S9 fractions) and single recombinant enzymes. Analysis of UGTs and SULTs isoform specific metabolism is performed using single recombinant enzymes, allowing to calculate kinetic parameters (Vmax, Km and Cli). The tissues distribution of the isoforms, mainly for UGTs, shows marked difference among the organs evidencing a potential extrahepatic metabolism relevance (Kasteel et al., 2020). Furthermore, in literature are reported data about age- and genotype-dependent variability in the protein abundance and activity of UGTs human liver (Bhatt et al., 2019; Kasteel et al., 2020), the isoform-specific metabolism may inform considerations of possible different susceptibility of the population to toxic effects.

Ultimately, all kinetic data will be used as an input in PARC for developing/enhancing physiologically based kinetic (PBK) models for each BPA alternative, including apparent (mass balance and radio-HPLC profiling studies in human liver models), and specific enzymatic kinetic parameters Vmax, Km and CLi constants of the single Phase I and II enzyme isoforms and human sub-cellular fractions of different organs.

## 3 Integration and prioritization logic

Data generated across toxicological domains will be interpreted within a screening-level weight-of-evidence framework designed to support prioritization of BPA alternatives for further investigation. Early-tier NAM assays serve as screening tools to identify mechanistic alerts, including endocrine activity, neurodevelopmental perturbations, immune modulation, genotoxic and non-genotoxic signals, or bioactivation potential. Substances showing consistent biological activity across multiple assays or endpoints may subsequently be prioritized for higher-tier targeted investigations, including advanced *in vitro* models, TG + studies, or refined exposure-based assessments.

Metabolism and kinetic data generated within the WP5 framework support the translation of *in vitro* findings into PBK modelling efforts conducted across PARC, enabling improved interpretation of internal exposure relevance. Through this cross-endpoint integration, the resulting evidence primarily provides screening-level mechanistic support for modeling, AOP development, and the design of follow-up studies, ensuring that prioritization decisions are guided by converging mechanistic signals across biological systems rather than single-assay outcomes. This approach supports regulatory evaluation processes and contributes to minimizing the likelihood of regrettable substitutions among BPA alternatives.

## 4 Conclusions

In the context of this coordinated tiered testing strategy using NAM-based batteries, WP5 partners are currently finalizing the presented tests. Integrated analysis of the generated datasets and publication of detailed experimental results are expected during the first quarter of 2027. Importantly, several NAMs included in this framework are also undergoing transferability and readiness evaluation activities, supporting future validation and regulatory uptake. This approach demonstrates how such efforts can be operationalized across multiple toxicological endpoints within a large regulatory research partnership. More particularly, it can help identify mechanistic alerts across endocrine, neurodevelopmental, immune, carcinogenic, and metabolic endpoints, thus supporting prioritization of BPA alternatives for further targeted investigation.

Importantly, the data generated within this program are expected to provide screening-level mechanistic evidence that can inform modeling efforts, AOP development, and the design of higher-tier investigations, thereby supporting regulatory evaluation processes. By combining harmonized experimental design, cross-endpoint integration, and PBK-informed interpretation, the PARC WP5 strategy proposes a scalable approach to minimize the likelihood of regrettable substitutions among structurally related chemical alternatives.

## Data Availability

The original contributions presented in the study are included in the article/supplementary material, further inquiries can be directed to the corresponding author.
